# Towards an extensive set of criteria for safety and cyber-security evaluation of cyber-physical systems

**DOI:** 10.12688/openreseurope.16234.1

**Published:** 2023-12-28

**Authors:** Aleš Smrčka, Behrooz Sangchoolie, Emanuele Mingozzi, Jose Luis de la Vara, Marie Farrell, Raul Barbosa, Cem Baglum, Ugur Yayan, Salih Ergun, Alper Kanak

**Affiliations:** 1 Brno University of Technology, Brno, Czech Republic; 2 RISE Research Institutes of Sweden, Borås, Sweden; 3 E.S.T.E., Ferrara, Italy; 4 Universidad de Castilla-La Mancha, Ciudad Real, Castile-La Mancha, Spain; 5 The University of Manchester, Manchester, England, UK; 6 Universidade de Coimbra, Coimbra, Coimbra District, Portugal; 7 Inovasyon Mühendislik, Eskisehir, Turkey; 8 Eskisehir Osmangazi University, Eskisehir, Turkey; 9 ERARGE, Ergunler, Isparta, Turkey

**Keywords:** criteria, evaluation, verification, validation, safety, cybersecurity, cyber-physical system (CPS)

## Abstract

Verification and validation (V&V) are complex processes combining different approaches and incorporating many different methods including many activities. System engineers regularly face the question if their V&V activities lead to better products, and having appropriate criteria at hand for evaluation of safety and cybersecurity of the systems would help to answer such a question. Additionally, when there is a demand to improve the quality of an already managed V&V process, there is a struggle over what criteria to use in order to measure the improvement. This paper presents an extensive set of criteria suitable for safety and cybersecurity evaluation of cyberphysical systems. The evaluation criteria are agreed upon by 60 researchers from 32 academic and industrial organizations jointly working in a large-scale European research project on 13 real-world use cases from the domains of automotive, railway, aerospace, agriculture, healthcare, and industrial robotics.

## 1 Introduction

Guaranteeing the safety of cyber-physical systems is becoming quite demanding, especially since these systems are increasingly facing security threats and attacks ^1^. These systems should go through rigorous verification and validation (V&V) processes before being used by the end users. Verification refers to the process of evaluating a system or component to determine whether the products of a given development phase satisfy the requirements imposed at the start of that phase, while validation refers to the process of providing evidence that the system, software, or hardware and its associated products solve the right problem (e.g., correctly model physical laws, implement business rules, and use the proper system assumptions), and satisfy intended use and user needs ^2, 3^.

V&V methods and tools aim to be effective and comprehensive, dealing with security challenges, safety-related implications, automation, and integration with the product life-cycle processes. V&V processes vary considerably for different types of System Under Evaluation (SUE), priorities of system requirements, severity and criticality of developed features, and the number of available resources (including but not limited to software tools for verification, their licenses, and hardware testbeds). There is no single metric that can be used to conduct a simple comparison of the different approaches to V&V and their complex characteristics, in order to help to select the best combination of V&V technologies for cyber-physical system development. The selection of V&V approaches should take into account not just the safety and cybersecurity of developed systems but also the different aspects of V&V processes used within a typical product life-cycle.

In this paper, we focus on the evaluation of V&V technologies and their impact on the safety and cybersecurity of cyber-physical systems. We provide an extensive list of evaluation criteria from which to choose when evaluating cyber-physical systems and the V&V processes used in their development.

The rest of the paper is organised as follows: The next section discusses the rationale behind the evaluation of different V&V activities, the categorisation of criteria, and the overview of the practical usage of evaluation criteria in real-world use cases. Section 3 and Section 4 list evaluation criteria for safety and cybersecurity aspects as well as criteria for measuring the impact of V&V technologies on time and cost of system development and evaluation. Section 5 presents an evaluation of the applicability of the criteria by reporting quantitative results obtained when they are used. The paper is concluded in Section 6.

## 2 Evaluating verification and validation activities

Product life-cycles ^4^ (especially for cyber-physical systems) typically consist of engineering phases starting with requirement specification, functional analysis and design, development, verification, deployment, operation and maintenance, and ending with the development evolution phase which closes the cycle. No matter what methodology the development teams follow, either waterfall-like or some kind of agile approach, every methodology includes feedback from V&V activities.

It is a well-known fact that shift-left strategy ^5^, which moves quality control to the earlier phases, can reduce the time and cost spent on fixing the design faults. However, when a complex combination of V&V techniques is used, it is difficult to measure the reduction properly. Moreover, it may be difficult to incorporate V&V technologies in the early phases of design because the technologies (i) might not be compatible with the implementation specifics of the target cyber-physical system, (ii) might not be suitable for the integration with other technologies used in the development phase, (iii) or even might not meet the skills of the development team.

V&V activities in product life-cycle phases include, among others, requirements analysis, design analysis, modelling and simulation, test selection and preparation, test execution, and measurement and reporting. Evaluating such activities cannot be done with a single metric—several aspects must be taken into account, for instance, the performance and accuracy of the results from the activity (how much it brings value), the cost of the activity (in terms of time, effort, or money), or the overall impact of the use of the activity on the whole product life-cycle. Moreover, if a metric is used as a single measure of the quality of V&V, the result may contradict the result from the measurement of another metric. For instance, less time spent on verification might express better efficiency (quality) of the V&V process but can also indicate that the process is not thorough enough, e.g., because the number of executed tests is small. Better coverage of test items by a large test suite may lead to thinking that the system has been checked properly but it does not say anything about the number of fulfilled or violated requirements. A larger test suite also often increases the effort for test creation, time of test execution, and even the cost of finding and fixing a bug.

There are quite a number of different quality metrics which describe in specific terms what is to be measured to provide sufficient data for decision-makers about developed products and processes behind the development. These metrics have already been documented and adopted by different standards but, in most cases, they do not take into account the applicability of the selected metrics. We have analysed 13 different real-world use cases based on the sample cyber-physical systems that are actually being used in six domains of application: automotive, railway, aerospace, agriculture, healthcare, and industrial robotics. A brief overview of the use cases is in Table 1; more information about the use cases can be found in ^6^. These cyber-physical systems are automated and partly include AI-based decision-making systems, which makes safety and cybersecurity their primary concern.

**Table 1.** Overview of 13 real-world use cases of cyber-physical systems upon which evaluation criteria have been identified. TRL represents the technology readiness level of developed/improved cyber-physical systems under evaluation.

Use cases	Domain(s)	TRL
Intelligent Traffic Surveillance	Automotive	4
Intelligent Traffic Surveillance	Automotive	5
Radar system for ADAS	Automotive	4
Human-Robot-Interaction in Semi-Automatic Assembly Processes	Industrial Robotics/Automation	5
Aircraft engine controller	Aerospace	5
Agriculture robot	Agriculture	3
Human-Robot Collaboration in a disassembly process with workers with disabilities	Industrial Robotics/Automation	4
Neuromuscular Transmission for muscle relaxation measurements	Healthcare	4
Autonomous train operations	Railway	4
Safe function out-of-context	Railway/Multi-domain	2
Automated robot inspection cell for quality control of automotive body-in-white	Industrial Robotics/Automation	4
Industrial Drives for Motion Control	Industrial Robotics/Automation	4
CardioWheel	Automotive/Healthcare	7

In the course of a large project (41 partners from 10 European countries) called VALU3S (ECSEL JU, 2020-2023) ^7^, the process of identification of verification and validation (V&V) criteria for the development and evaluation of cyber-physical systems has been established based on a collaboration between practitioners and researchers. The process was kicked-off in one of the project’s tasks, which had the objective of planning for the evaluation of the project and its demonstrators. As a result of the activities performed in this task, a report was published ^8^, which consisted of an initial set of evaluation criteria and outlined plans by various partners for the application of these criteria in their respective domains. The report was generated as a result of the coordination meetings organised that were an integral part of this phase consisting of industry and research partners as well as providers of 13 project use cases. The participants of these meetings were selected collaboratively, paying special focus on their relevance and roles in the project and their contributions to the criteria refinement process. The authors of this paper, in collaboration with all project partners, engaged in structured discussions that covered aspects such as product quality measurement, evaluation of development processes within companies, the potential for additional measurements, and the benefits of measured metrics. The process did not involve audio or video recordings of the meetings. Unfortunately, due to the confidentiality clause of the project’s consortium agreement, we cannot provide details of these discussions. The refined criteria, as presented in the paper, evolved from these collaborative efforts, with subsequent evaluations using these criteria publicly available in the project web repository ^9, 10^.

We then refined and related the metrics with the literature and existing standards and searched for commonalities among the domains. The refinement process of the metrics resulted in the selection of criteria which provide means for practical measurement of the development of cyber-security systems. We have concluded with two categories of practical evaluation criteria which are common across the use cases. The first category includes evaluation criteria that are suitable for the measurement of Safety and Cybersecurity (SC) attributes (see Section 3), whereas the second category focuses on the evaluation criteria for measuring the efficiency of V&V processes (see Section 4). The SC evaluation criteria take into account the following parameters: (a) safety/security requirements of the developed systems; (b) faults and attacks, their artificial injection into the system and detection and reaction by the system; (c) impact and prevention of incidents, attacks, and accidents; (d) AI/ML classification metrics; (e) accuracy and duration of authentication and cryptographic algorithms; and (f) simulation accuracy. The V&V evaluation criteria directly or indirectly express the spent time and cost of the V&V processes. In particular, they take into account the following parameters: (a) effort spent on requirements engineering; (b) number of test cases; (c) coverage of the test set; (d) time and effort for the test execution (including preparation and manual testing); (e) statistics about continuous development and integration; (f) effort for the security assessment; and (g) reliability of the V&V technologies.

## 3 Criteria for evaluation of cyber-physical systems regarding safety and cybersecurity

This section provides the list of criteria for evaluating the safety and cybersecurity of CPS; further referred to as SC-x (where x is the number of the criterion).

### 3.1 Error coverage (SC-1)

Error coverage, usually denoted by *c*, is defined as the conditional probability that a system recovers, given the occurrence of a fault ^11, 12^. In standards such as ISO26262, a similar metric is suggested to be used that is called *failure mode coverage* corresponding to the proportion of the failure rate of a failure mode of a hardware element that is detected or controlled by the implemented safety mechanism ^13^. Here failure mode is described as the nature of a failure, i.e., the way in which a program or system can fail. Similar to other metrics such as program vulnerability factor (PVF) ^14^, error coverage does not distinguish between different failure modes. However, in practice, silent data corruptions ^1^ (SDCs) are considered the most severe failure mode, because users will trust the system output in the absence of an error indication. This is because the erroneous outputs are generated with no indication of failure, making them very difficult to detect. Instead of the error coverage, some researchers have used error resiliency ^15, 16^ as the dependability metric where error resiliency is defined as the conditional probability that the system does not produce an SDC after a fault occurs and impacts the system state.

**Measured artefacts** The percentage of errors covered by a system could be measured by conducting fault injection experiments (these experiments are recommended in more than 10 assessment methods in the ISO26262 standard ^13^), by considering the following parameters and the formula:

- Total number of faults injected into the system [ *nInjections*].
- Number of cases where the system recovers after injection of faults [ *nRecovery*]
-
$ErrorCoverage=\frac{nRecovery}{nInjections}$

It is worth noting that cybersecurity attacks may be considered as a special type of fault that is human-made, deliberate, and malicious, affecting the hardware/software from external system boundaries and occurring during the operational phase ^17^. Therefore, error coverage and error resiliency could also be measured by taking the cybersecurity attacks into account in addition to hardware and software faults. Researchers utilised this analogy to measure the impact of cybersecurity attacks on systems resiliency ^18– 21^.

### 3.2 Number of safety/security requirement violations (SC-2)

Measuring the number of violated safety or security requirements that have been checked by runtime monitors, software testing, and/or formal verification is useful for comparing the effect of changes to requirements engineering, development, and verification processes. It is important to remember that the violation of security requirements can negatively impact a system’s ability to uphold its safety requirements ^22^. For example, a security violation in an autonomous vehicle could result in a critical situation where the AI algorithm can not recognise the vehicle in front and this may cause a crash resulting in the violation of related safety requirements ^23^.

Safety/security requirement violations may indicate inconsistencies or contradictions amongst requirements if any do exist. If such a case presents itself, it may be necessary to rework the requirements and/or rank the requirements so that the most important ones are addressed by the system or reach some compromise amongst requirements (e.g., ^24, 25^). Many international standards, including DO-178C in the aerospace domain ^26^, advocate a requirements-driven approach to development (supporting traceability from requirements to implemented systems) and a number of software tools have been developed to support the task of requirements elicitation, specification and management ^27, 28^. Recent techniques involve reducing ambiguities in natural language requirements so that they are precise by using a semi-structured and more formal syntax ^29^.

**Measured artefacts**

- Number of safety/security requirements [ *nR*]
- Number of safety/security requirement violations (this could also be considered as a percentage/ratio of the previous) [ *nV*].
-
$V=\frac{nV}{nR}$

Requirement violations can be detected by a number of verification activities including formal methods, simulation, testing and run-time monitoring. However, it is important that when violations are detected that the root cause of the conflict is identified and resolved. One way to reduce requirement violations from the outset is to follow a methodical requirements elicitation and specification process that involves formalising and slowly refining the requirements. This involves beginning with a high-level set of requirements that are gradually decomposed into more detailed, specific requirements ^30^. Typically, this kind of process would start with abstract natural language requirements and return to a larger set of formalised requirements. Here we can measure the following:

- Number of natural language requirements [ *nL*]
- Number of formalised requirements [ *nF*]

These numbers give an idea of the effort involved in removing ambiguities from natural language requirements and the formalised requirements can be used as direct input to other verification tools/techniques.

### 3.3 Number of malicious attacks and faults detected (SC-3)

This evaluation criterion measures the number of malicious attacks and faults detected in the SUT compared with the actual number of malicious attacks and faults that have been injected into the SUT, to reflect on safety and security aspects. The SUT would be considered safe and secure when the faults and attacks are correctly detected (there are attacks that could cause safety violations, similar to faults that could result in security violations). Requirements for the detection rates depend very much on the application and context of the SUT. Therefore, this shall be defined according to the risk assessment performed by the SUT (e.g, Hazard analysis and risk assessment in ISO 25119 ^31^ or threat analysis and risk assessment in ISO 21434 ^32^.

**Measured artefacts** Once the malicious attacks and faults are clearly defined, the following artefacts shall be measured:

- Number of detected malicious attacks [ *n _DetectedAttacks_*]
- Number of detected faults [ *n _DetecteFaults_*]
- Number of actual/injected malicious attacks [ *n _ActualAttacks_*]
- Number of actual/injected faults [ *n _ActualFaults_*]

Once the above measures are available, the safety and security of the system can be evaluated by comparing the number of detected measures with the actual/injected measures. For instance, the detection rate (DR) could be defined as follows:


$$DR_{attack}=\frac{n_{DetectedAttacks}}{n_{ActualAttacks}}$$

$$DR_{faults}=\frac{n_{DetectedFaults}}{n_{ActualFaults}}$$

### 3.4 Metrics to evaluate artificial intelligence/machine learning algorithms (SC-4)

Machine Learning (ML) is the area of Artificial Intelligence (AI) that mainly studies example-based supervised learning algorithms. The goal of ML is to gain insights from data and past experiences to make predictions for future scenarios. This involves fitting mathematical models to available data and then using those models to predict newly observed data. Data quality and preparation, along with model training, are key aspects of the ML pipeline. However, it is equally important to measure the performance of the trained models. If such models fail to achieve the necessary in-context performance, there might be safety risks for users in domains such as autonomous vehicles and health care.

There is no single absolute metric of the overall predictive power of a model such that one could rely upon this metric before deployment. Instead, one should consider multiple metrics to evaluate the ML models. Furthermore, specific domains may impose specific preferences, for example, measuring accuracy may suffice for many automotive applications, while healthcare scenarios most often require measuring sensitivity and specificity. Therefore, the use of distinct evaluation metrics is critical in ensuring that a model will operate correctly and optimally in production use.

**Measured artefacts** There are many metrics to evaluate AI/ML algorithms classified in different categories. Some of the most relevant ones are listed below:

- Classification metrics:
  – True positives (TP): Test results for which the model correctly predicts the presence of a characteristic class.
  – True negatives (TN): Test results for which the model correctly predicts the absence of a characteristic class.
  – False positives (FP): Test results for which the model incorrectly predicts the presence of a characteristic class.
  – False negatives (FN): Test results for which the model incorrectly predicts the absence of a characteristic class.
  – Accuracy: Measures observational error regarding the true value, with $Accuracy=\frac{TP+TN}{TP+TN+FP+FN}$ , equaling the number of correct predictions divided by the total number of test results.
  – Precision: Indicates the proportion of positive identifications that are correct, as yielded by $Precision=\frac{TP}{TP+FP}$ .
  – Recall (or sensitivity): Indicates the proportion of actual positives that are correctly identified, as yielded by $Recall=\frac{TP}{TP+FN}$ .
  – Specificity: Indicates the proportion of actual negatives that are correctly identified as such, given by $Specificity=\frac{TN}{TN+FP}$ .
  – F1 score: Combines precision and recall into one metric by taking the harmonic mean of those two.
  – Classification threshold: The result of an ML model is often a probability or a score that needs to be converted into a final class label. To this end, a parameter named “classification threshold” or “decision threshold” is introduced as a means to control which output values are mapped onto which classes. Fine-tuning this threshold may for instance improve the balance between true positives and false positives.
  – Receiver Operating Characteristic (ROC) curve: The ROC curve is a plot of the performance of a classification model at different classification thresholds. Namely, the ROC curve plots the sensitivity against the false positive ratio for varying thresholds.
  – Area Under the Curve (AUC): The AUC is a measure of the area between the ROC curve and the horizontal axis. The AUC is often used for model comparison.
- Regression metrics:
  – Mean Squared Error (MSE): The MSE is the mean or average of the squared differences between predicted and actual target values, measured using a given dataset.
  – Mean Absolute Error (MAE): The MAE is defined as the arithmetic average of absolute errors, therefore measuring the average magnitude of errors in a set of predictions disregarding their direction.
- Ranking metrics:
  – Reciprocal Rank (RR): The RR information retrieval measure is the reciprocal of the rank at which the first relevant document was retrieved. The Mean Reciprocal Rank (MRR) is the average RR across a sample of queries, which is a statistical measure suitable for evaluating information retrieval processes that return ordered results.
- Computer vision:
  – Intersection over Union (IoU): It is defined as the overlapping area, between the predicted bounding box and the ground-truth bounding box, divided by the area of the union of those two bounding boxes.
- Beyond statistical metrics: For some critical applications it may be relevant to verify if a model systematically fulfils some properties. In such cases, verification techniques may be applied to deduce whether the model always guarantees such properties, regardless of the input.

For instance, in a surveyed automotive use case on driver’s drowsiness detection, machine learning algorithms are trained for each individual driver, by acquiring ECG (electrocardiogram) signals during driving periods to classify drivers as drowsy or alert.

### 3.5 Potential impact of incidents and attacks (SC-5)

This criterion defines different levels of impact that the attack might have on the attacked system, classifying the impact of attacks depending on malicious effects on the attacked system and on the actions that are required to make the system operative again.

**Measured artefacts** The number of service and system interruptions to restore the service and the system from the effects of the attack, the number of total and partial damages to the attacked system or any external object and/or affected people. The potential impact of incidents and attacks is evaluated by using the following scale with increasing impact:

- Level 0: Service interruption which does not require human intervention to be solved. The interruption does not cause any damage.
- Level 1: Service interruption which requires human physical or remote intervention to be solved. The interruption does not cause any damage.
- Level 2: System partial damage which requires human physical maintenance intervention to be fixed. The damage does not cause any harm to external objects or people.
- Level 3: System total damage which cannot be fixed by human physical maintenance interventions but requires a complete system replacement. The damage does not cause any harm to external objects or people.
- Level 4: System total or partial damage which can potentially cause harm to external objects and people within the surrounding environment of the attacked system.
- Level 5: System total or partial damage which can potentially cause harm to external objects and people even not physically located in the surrounding area of, or related to, the attacked system.

The above classification is an application of the definition of impact rating of NIST SP 800-30 ^33^.

### 3.6 Metrics to evaluate cybersecurity (SC-6)

The development of secure systems needs to be validated using relevant metrics to judge the quality of cybersecurity, so that over several development iterations, the number of, e.g., identified threats are reduced. Hence, this metric aims to help developers prioritise the threats to be treated to improve the cybersecurity of the product.

**Measured artefacts ** Some of the metrics that can fall under this criterion are as follow:

- Number of threats (grouped by severity). Number of threats that may have hazardous safety implications.
- Number of attack paths (grouped by feasibility, e.g., as described in ISO/SAE 21434, Annex G ^32^).
- Number of threats successfully exploited during testing / total number of threats identified during analysis (e.g., by using penetration testing tools).
- Number of attack paths successfully exploited during testing / total number of attack paths identified during analysis (e.g., by using penetration testing tools).
- The amount of time in which the system under test is available after the initiation of a cybersecurity attack (also referred to as Survivability ^34^).
- Optionally, indirect measurements (through other metrics) of the effects of redundancy of different parts of a system on cybersecurity (if it improves or weakens the security).

Among the use cases in which these metrics are used, the evaluation of car teleoperation must consider the results from threat analysis and penetration testing as these methods are in compliance with automotive standards.

### 3.7 Number of prevented accidents (SC-7)

This criterion aims at assessing the situations which could lead to an accident related to the safety of a system. The criterion is used to verify that safety mechanisms prevent faults from leading to an accident ^35^. There can be mainly two approaches to measure the number of prevented accidents: (i) to analyse a model of a system and provide a detailed report about conditions of possible accidents; (ii) to experimentally evaluate the system and report each accident. These reports are then used during the implementation and evaluation of appropriate safety mechanisms.

The number of traffic accidents could also be a useful measure when analyzing traffic statistics. This way, a reasonable level of risk of one accident per X km (or even per Y hours) could be derived as suggested by ISO 21448 ^36^. Moreover, according to this standard, this measure is also important as, from the point of view of users of automated driving functions as well as society, the desired behaviour of such functions is "never has an accident or causes an accident". However, there might be a gap between the desired behaviour and implemented behaviour and specified behaviour (see Figure A.17 of ISO 21448 ^36^). Note that this metric would also need to be complemented with other metrics such as the severity of accidents for those accidents that could not be prevented.

**Measured artefacts**

- Number of correctly prevented accidents when the SUE is equipped with a safety mechanism [ *nPA*]
- Total number of accidents that could have occurred given that no safety mechanism is in use [ *nA*]
- Percentage of prevented accidents: $PA=\frac{nPA}{nA}$

### 3.8 Authentication accuracy and time applied to human users and components (SC-8)

This evaluation criterion deals with the cybersecurity of systems interacting with human users. Although there are several approaches to deal with authentication ^37^, this criterion focuses on two main sub-areas of cybersecurity: (i) active authentication of system components or nodes in general, at certain time intervals to verify that each component is not under attack (active authentication), (ii) role-based access module for user authentication against unprivileged attempts to access a system.

In all application cases, the authentication of users and nodes is an indispensable requirement. For instance, in fleet traffic management authentication of drivers is needed and each driver should be associated with an authenticated vehicle (node in city traffic). In another example, workers are tracked by a smart system either to monitor their efficiency or protect them against accidents. For the sake of better worker safety, workers’ functions and their relations with the physical environment and industrial nodes, e.g. robotic systems should be modelled. In such a model, the authentication of both workers and the interacted physical settings should be performed with high accuracy and throughput, so that the accountability, security, safety and privacy requirements can be met.

**Widely preferred measured artefacts (acceptance criteria)**

- Person Authentication Criteria:
  – Authentication accuracy rate to be *>* 99%.
  – Authentication duration in seconds to be *<* 2 *s*.
- Node Authentication Criteria:
  – Authentication accuracy to be *>* 99.9%.
  – Authentication duration in seconds to be *<* 1 *s*.
- The authentication process is to be completed within a maximum of 5 seconds at the latest.
- In the case of a system using biometric authentication, False Acceptance Rate (FAR), False Rejection Rate (FRR), Equal Error Rate (EER, where FAR = FRR), and AUC values can be used alternatively. Generally, EER is expected to be *<* 1%.

Note that, the evaluation criteria for authentication is two-folded, i.e. person and node (or thing) authentication. Standardisation activities are so diverse that both national and international standard organisations deal with multi-factor authentication of a person on one hand, and authentication of system components in heterogeneous and distributed networks (e.g. IoT networks) on the other hand. ISO 27002 ^38^ specifies the secure authentication protocols to protect login portals against unauthorised access attempts. ISO/IEC 27553 ^39^ defines the security and privacy specifications of biometric authentication techniques used in mobile applications. In recent years, Fast Identity Online (FIDO) standards ^40^ have gained importance as these standards are supported by companies like Google to make user authentication easier and more secure in online applications. For the authentication of things in complex industrial and IoT systems, the standardisation activities are still evolving. ISO/IEC 9798 ^41^ and 15408 ^42^ standards partly cover the security of IoT hardware and related software with the support of standards in cryptography, e.g. ISO 27002 ^38^. In this study, FIDO and ISO/IEC 15408 are followed as the main standards during the design and implementation of authentication schemes.

### 3.9 Randomness and cryptographic algorithm strength (SC-9)

This criterion aims to ensure the entire security of a cyber-physical system, covering the end nodes and central mechanisms, through highly secure cryptographic backends. Cryptographic algorithms are indispensable to secure any data generated, shared and exchanged within a system especially to improve cyber resilience against attacks like man-in-the-middle, sniffing, and denial of service. A typical cryptographic backend is roughly composed of the cryptosystem architecture, cryptographic algorithm and key generation mechanism. According to Kerckhoffs assumption ^43^ although cryptographic algorithms are crucial and must be kept secret, the strength of a cryptosystem is highly dependent on the cryptographic key generation mechanism. Here, randomness plays a critical role because the cryptographic keys should not be predictable and they must be generated by reliable and robust hardware-based truly random number generators ^44^. Thus, SC-9 is defined as a key criterion with the following artefacts to assess the resilience of the backend cryptosystems that are widely used in cyber-physical systems.

**Measured artefacts**

- Passing the 4-step test routine which is composed of analysing the vulnerabilities of a cryptographic key generator by testing (i) true randomness, (ii) unpredictability of the bit streams, (iii) irreproducibility of crypto-keys, and (iv) robustness analysis of the cryptosystem. The randomness test results are then presented in terms of pass rates being higher than 98%. It is expected that the true randomness, unpredictability, and irreproducibility criteria are met cordially (passing all criteria is a necessary condition, while the performance criteria are sufficient conditions).
- Percentage of the functionality of both symmetric and asymmetric cryptographic algorithms within their particular context (encryption and decryption working properly) in terms of algorithm speed (e.g., AES speed less than 1 Gbit/sec; RSA-512 minimum number of operations per second less than 20; SHA-512 speed minimum 3.5 Gbit/Sec). The functionality of each algorithm can be measured by the success rates of self-tests, for instance, the encryption of a pre-defined test data (e.g., 5 MB) that should be executed within a reasonable time (e.g., *<* 0.1 *s*).
- Encryption/Decryption duration in seconds (expected: *<* 1 *s*)

In industrial robotics, this criterion has been addressed to improve the cyber-physical resilience of an automotive body-in-white quality inspection system against malicious or unintended access to or use of camera recordings and system data. In such a use case, the strength of the cryptographic backend is crucial because the evaluated cyber-physical system generates critical data that should be protected against attackers. For instance, the quality inspection system generates results about the resilience of automotive body parts. If the quality analysis results are manipulated, this may cause serious manufacturing and safety problems. Moreover, as automotive manufacturing processes are getting multi-stakeholder, sharing data among the collaborating organisations by considering secure storage and transmission is indispensable. Hence, this criterion is needed to assure the trusted encryption of the quality inspection data.

The standardisation activities in cryptography face many challenges as there exists a lack of consensus among national and international standard organisations. The effectiveness of standards are questioned as there has been an everlasting fight between attackers and defenders ^45^. The widely adopted international standards for encryption algorithms are listed in ISO/IEC 18033 ^46^ series including the asymmetric schemes, block and stream ciphers. For hashing ISO/IEC 10118 ^47^ standard is generally accepted.

In all these cryptographic standards, random number generation is strictly addressed. ISO/IEC 18031 ^48^ and 18032 ^49^ are two mainstream standards for random bit and prime number generation. Besides, NIST 800-22 Randomness Test Suite ^50^, which is also followed in this study, has become a defacto technique to measure true randomness. On the other hand, the strength of a cryptographic system can be measured in many ways. The most widely adopted technique is based on the Common Criteria Evaluation Framework that relies on the ISO/IEC 15408-5:2022 ^42^ evaluation criteria for information technology security (as applied in this study). This standard presents different levels of security, namely the Evaluation Assurance Level (EAL), where EAL4 or higher levels present methodically designed, tested and reviewed security systems where information security is guaranteed.

### 3.10 Software fault tolerance and robustness (SC-10)

Robust and fault-tolerant software is expected to function correctly, remain stable and avoid service failures in spite of eventual errors. There exists a subtle distinction between fault tolerance and robustness: the former aims to avoid failures in the presence of faults in general, whereas the latter aims to ensure the correct reaction to the specific class of external faults ^17^.

An error is a deviation of a component’s state from what is considered correct and that may lead to a failure. An error may remain internally latent if it does not reach the service interface to cause a failure; a detected error is indicated by an error signal or an error message; undetected errors may propagate to the service interface and cause failures. Errors are caused by faults that are activated ^17^. Error coverage (see Section 3.1) is a related concept because it can be achieved through fault tolerance and robustness means.

Consequently, software fault tolerance and robustness are verified by means of different testing methods, including but not limited to fault injection. The fault injection method is based on deliberately inserting faults or errors into systems to study their ability to handle inefficiencies or malfunctions. Representative fault models for software fault injection have been widely researched ^51^, also specifically addressing the emulation of security vulnerabilities ^52^ and robustness testing ^53^.

**Measured artefacts** The fault tolerance and robustness can be measured by a number of invalid software conditions the system can handle either by recovering from an error state or by denying the input conditions. It is indicated as the portion of defined software faults while providing its specified functionality. Different measures can be used for invalid conditions that are application specific, e.g., a number of invalid inputs, a number of faulty software components, and unexpected timing. Fault tolerance and robustness (FTR) can be evaluated as follows:


$$FTR=\frac{\text{denied}\;\text{or}\;\text{correctly}\;\text{avoided}\;\text{conditions}}{\text{number}\;\text{of}\;\text{invalid}\;\text{conditions}}$$

For instance, in a surveyed agriculture use case on a robotic lawn mower, an unmanned ground vehicle, communication disturbances between control units are expected, for example, due to noisy wireless communication between a remote operator and the ground vehicle. As such, invalid conditions are expressed as unreliable communication link parameters. Forthcoming standards on autonomous systems are being developed to guide developers on fail-safe design, and these include the IEEE P7009 standard ^54^.

### 3.11 Simulation-level system robustness (SC-11)

This criterion is similar to the software fault tolerance robustness criterion (see Section 3.10), but the assessment is not restricted to software and instead can potentially include, e.g., model-in-the-loop or hardware-in-the-loop components. ISO 5055 ^55^ standard was used to assess the quality of the tested software by identifying and counting instances of poor architectural and coding practices in the source code that could result in high operational risks or unnecessary costs. This standard aims to ensure the safety of the system by providing a suitable structure for both humans and computers to use, using a quality model for safety-critical systems. The model is based on ISO/IEC 25010 and takes into account both the dynamic and static properties of the computer and software. Simulation-level system robustness is an important criterion for verifying and validating safety-critical systems. For example, the system Robustness calculation could be used to determine the robustness of the system for the simulation of robotic arms.

**Measured artefacts** Similarly, the fault tolerance robustness can be measured by considering various fault types that may occur at assignment, algorithm, or timing level, and can be evaluated by the following formula:


$$SystemRobustness=\frac{\text{number}\;\text{of}\;\text{successful}\;\text{simulation}\;\text{runs}}{\text{number}\;\text{of}\;\text{fault}\;\text{injection}\;\text{scenarios}}$$

### 3.12 Number of attack/incident typologies examined (SC-12)

Each method/tool is designed to deal with certain hazards or threats: there are tools specifically dedicated to the analysis of only one single type of attack/incident, while others can cover a large set of typologies, including both safety and security issues. Attack typologies define the cyber attacks, also defined in ^56^, while incident typologies define the type of hazard that causes an incident ^57^. The higher the number of attack/incident typologies which are considered, the more exhaustive the treatment will be, but at the same time, the level of detail could be lower when larger sets are included.

This criterion expresses the number of attack or incident typologies the method or tool is capable of dealing with, giving a useful indication of its functionalities and, indirectly, about its level of detail.

**Measured artefacts ** The number of attack/incident typologies examined by a certain method/tool.

### 3.13 Scene simulator quality (SC-13)

Virtual environments provide a presentation of sensory information mimicking a real-world physical environment. Simulators coupled with a virtual environment enable experiments on cyber-physical systems that are otherwise hard to achieve. Increased functionality and quality of the simulators will gain more opportunities to simulate complex and possibly harmful or expensive events. Test scenarios generated for sensing systems, e.g., camera-based monitoring or Advanced Driver Assistance Systems (ADAS), should correspond to physical world situations to achieve the best accurate results compared to the field experiments. The higher the quality of the simulator, the higher the level of safety assurance we get. This criterion focuses on the quality of simulators which provide 3D scene test data (visual or another type, e.g., point cloud for LIDAR) for automated cyber-physical systems. There are several standards, e.g., ISO/IEC 10641 ^58^, ISO/IEC 25010 ^59^, which provide a framework for evaluation of models used for the simulation of developed systems, but they do not specifically mention the use of selected metrics for 3D scenes and 3D virtual environment simulators.

**Measured artefacts** The quality of such simulators should be expressed by (i) the accuracy of the simulated sensor output, (ii) scene quality, and (iii) simulator environment functionality as described below:

- Accuracy of the simulated sensor output generated by a simulation environment should be compared with real sensor data from a controlled and virtually replicated environment to verify the simulator output. Examples of measured artefacts are Fréchet inception distance ^60^ (a metric for evaluating the quality of generated images which compares the distribution of generated images with the distribution of real images; lower the better), spatial resolution and density (higher the better), and bit depth difference of the imaging systems and the simulator (lower the better).
- Scene quality includes metrics to track visual quality and model resolution (e.g. polygon counts and real-time rendering performance; the higher, the better).
- Simulator environment functionality is expressed with the number of supporting functionalities/modules for scenario generation that are available by the simulator (higher the better).

## 4 Criteria for measuring the efficiency of V&V processes/activities

The section provides the list of criteria for evaluating V&V processes and their activities. The criteria are further referred to as VV-x (where x is the number of the criterion).

### 4.1 Time of test execution (VV-1)

Given different versions of test sets with very similar test coverage, the criterion aims at comparing the execution time of the test sets and the number of faults/attacks in the test sets. This criterion will show if and how a new test set will be optimized w.r.t. used methods, improved tools, and available resources. Testing is a key activity to confirm that a critical system’s behaviour is adequate ^13, 26^, can require the execution of several testing types depending on the testing objective, environment and elements targeted, and can require considerable time.

**Measured artefacts** Test execution time.

### 4.2 Coverage of test set (VV-2)

This criterion deals with measuring how much software/hardware test coverage items have been covered by a test set (set of test cases, also known as test suite). Examples of these items are lines of code, branches, faults, and attacks depending on the selected test design technique. Note that increased coverage means increased trust in the analysed system.

**Measured artefacts** Examples of measured artefacts are (i) the number of test coverage items covered by the executed test cases, and (ii) the total number of test coverage items identified by the test design technique.

Note that there are different metrics of a source code defined by so-called structural coverage criteria. For instance, ISO 26262 ^13^, and DO-178C ^26^ recommend all statements or branches to be covered for non-critical systems, but highly recommends full *Modified condition/decision coverage* ^61^ for critical systems.

### 4.3 Number of test cases (VV-3)

Using this criterion, one can quantify a test set, proving that a reduced number of test cases is able to ensure desired quality (coverage), e.g., in combination with measurement of error coverage, number of safety/security requirement violations, and number of malicious attacks and faults detected. In ISO 21448 ^36^, it is, e.g., suggested to use known scenarios as a basis for constrained random generation of tests of new scenarios, so the testing coverage space is increased incrementally. This in turn would result in the number of test cases also increasing incrementally instead of exponentially.

Through conducting analyses such as test space pruning, one could reduce the number of test cases contributing to a more cost-efficient evaluation of SUE. For V&V methods such as fault injection (fault injection is recommended by more than 10 assessment methods in the ISO26262 standard ^13^), reduction of the test space is necessary as this method, in general, comes with significant evaluation time and cost related issues. Fault injection techniques, therefore, are equipped with different types of analysis, facilitating the reduction of the test space. Examples of these analyses are: inject-on-read ^62– 65^, inject-on-write ^64– 66^, code-slicing ^67^, fault list collapsing ^68, 69^, error space pruning ^65, 70– 74^, and post-injection analyses ^75– 78^.

**Measured artefacts** An example of measured artefacts is the total number of test cases required for system evaluation. This artefact could then be used to measure the reduction in the size of the test space by measuring the following:


$$\left(1-\frac{\text{size}\;\text{of}\;\text{the}\;\text{test}\;\text{space}\;\text{after}\;\text{using}\;\text{a}\;\text{test}\;\text{space}\;\text{pruning}}{\text{size}\;\text{of}\;\text{the}\;\text{test}\;\text{space}\;\text{prior}\;\text{to}\;\text{incorporation}\;\text{of}\;\text{any}\;\text{test}\;\text{space}\;\text{pruning}}\right)$$

### 4.4 Effort for test creation (VV-4)

This criterion deals with the estimation of effort for deriving and/or maintaining test suites, e.g., for fault injection and runtime verification campaigns (manual design vs model-based generation) ^79^. Test planning should consider the time spent on test designs; for instance, ISO 29119 standard ^80^ puts importance on the estimation of effort and elapsed time while designing tests. The criterion is normalised to the number of test cases in a test suite so it can be used to estimate an effort for the creation of new test cases.

**Measured artefacts** An example of measured artefacts is the time (Hours or Person-months) for test creation and test maintenance over the total number of test cases (the lower, the better):


$$\frac{\text{time}\;\text{for}\;\text{test}\;\text{creation}\;\text{and}\;\text{maintenance}}{\text{number}\;\text{of}\;\text{test}\;\text{cases}}$$

### 4.5 Joint management of SCP requirements (VV-5)

In real-world cyber-physical systems, many kinds of requirements must be considered and often addressed jointly to deal with requirement interdependencies. Next to the analysis of purely functional requirements, SCP requirements and their influence on each other need to be considered (e.g., see ^20, 72, 81, 82^ for the interrelation between safety and security). Often, safety, cybersecurity and privacy are treated separately by domain experts, which bears the risk of missing important effects of solutions in one quality attribute domain (SCP) on another. To minimize risks and costs and similar to what has been done in previous studies ^19, 20, 82– 85^, the potential impact of SCP requirements on the design (and later stages in the Product Life Cycle) must be analysed early with the management flow for joint SCP requirements analysis.

In standards such as ISO 21434 ^32^, it is indicated that an organization shall identify disciplines related to, or interacting with, cybersecurity and establish and maintain communication channels between those disciplines in order to (a) determine if and how cybersecurity will be integrated into existing processes, and (b) coordinate the exchange of relevant information. The disciplines mentioned in this standard are information technology security, functional safety, and privacy. A requirement as such motivates having metrics connected to joint management of SCP requirements, where as part of this management, one could also identify cybersecurity requirements conflicting or competing with functional safety requirements.

**Measured artefacts**

- Number of joint (combined) SCP requirement engineering (management) techniques (or tasks) that are part of target systems’ product life cycle and jointly treat more than one SCP requirement.
- Number of test cases that deal with more than one requirement type. This could also include, e.g., the introduction of a cybersecurity attack into a system while measuring its impact on system safety ^18, 19^, something that is also indicated in ISO 21434 ^32^ (see Chapter 15.5.2 of the standard).

### 4.6 Cost of finding and fixing a coding bug (VV-6)

The criterion aims only at failed tests ^79^. The testing process does not end with the execution of the test suites. If some tests fail, it is up to practitioners to start debugging and finding and fixing a bug. It is not necessary to test a single test coverage item separately. Some tests focus on test execution reduction by targeting more than one test coverage item in a single test execution. ISO 29119 ^80^ recommends considering debugging times while deriving tests. Tests which combine several test items could also increase debugging times as the test scenario will be complex and hard to analyse. In other words, simple tests relatively reduce the time needed for bug fixing as several bugs could be manifested in a single execution of the test suite.

**Measured artefacts** Test preparation time, test execution time, finding and fixing time, and the number of bugs. These artefacts can then be used to calculate the cost of a bug found in-house:


$$\text{test}\;\text{preparation}\;\text{time}\;\text{+}\;\text{test}\;\text{execution}\;\text{time}\;\text{+}\;\frac{\text{finding}\;\text{and}\;\text{fixing}\;\text{time}}{\text{No}\;\text{of}\;\text{bugs}}$$

### 4.7 Development quality statistics (VV-7)

Evaluating development and code statistics is a valuable tool for keeping track of software functionality and quality. Preferably the statistics should be evaluated after every software change, e.g., code updates, or at scheduled times, e.g., periodically every night. Evaluation might be connected with running regression tests and using the results as part of the statistics.

**Measured artefacts** There are several metrics to be measured, but the following list is a strong recommendation covering the fundamental needs of software quality ^79, 86^; the selection depends on tools available to measure data automatically:

- Total number of regression tests
- Test execution coverage—percentage of the regression tests run out of a total number of regression tests per commit or feature branch merge (in a version control system)
- Test execution time
- Defect rate percentage—percentage of tests that failed out of a total number of test cases executed
- Defect coverage—percentage of previously detected defects that are covered by regression tests
- Code review—ratio of commits which have been reviewed to the total number of commits
- Productivity—new lines of code per man-day

### 4.8 Effort needed for test (VV-8)

This evaluation criterion is used to measure the effort (e.g., person-hours) required to perform a test on a system. This encompasses the entire process of doing a test, from the dataset generation, execution of the test cases and the validation of the results. The effort can be measured by considering the number of people involved in the work and the number of hours needed to complete a task. This measure is especially useful to compare the effort spent on manual work versus automated work ^79^. ISO 15288 ^87^ includes safety standards that are used in V&V methods, such as the fault injection method, to gather information about the results of effort measurement in various situations.

**Measured artefacts** Total person-hours cost, i.e., the total person-hours per task, is obtained by multiplying the number of people assigned to a task normalised to Full-Time Employee (FTE) by the total completion time.

### 4.9 Service actions needed (VV-9)

The number and complexity of service actions (human interactions including but not limited to updates and fixes because of hardware/software faults) needed after deployment of the system to the field. This can include the first installation of the system, on-site debugging and tuning of the configuration during the first phases of operation (i.e., weeks usually), and regular service checks (e.g., monthly, or quarterly) or demanded actions. In case of serious problems, the product can be returned to development in order to be modified. Such actions are typically required by assurance and engineering standards such as ISO 26262 ^13^ and DO-178C ^26^.

**Measured artefacts** The quality of a product can be measured during its life cycle as the number of service (maintenance) actions and effort per task. For the sake of evaluation purposes, only selected relevant actions should be taken into account. For instance, this evaluation criterion is applied to the development process of intelligent traffic surveillance systems based on cameras and radars that are connected to the cloud. Manual service actions of a stationary camera are time-demanding and require careful planning.

### 4.10 Cost and time for work on the certification process and functional safety (VV-10)

Successful certification of critical systems demands compliance with applicable technical guidelines and standards of the specific application domain, such as DO-178C in aerospace ^26^, and ISO 26262 in automotive ^13^. System assurance and assessment must consider several costly and time-consuming activities, such as (i) hazard and risk assessment, (ii) compliance management, (iii) evidence management, and (iv) assurance case development.

For instance, in the scope of the automatic system for measurement of neuromuscular transmission for muscle relaxation, this criterion is used to study the extent to which advanced methods and tools for compliance management, system artefact quality analysis, and traceability management can reduce the cost and time for system certification.

**Measured artefacts**

- Number of tasks to be performed for certification purposes and estimated effort (time and costs) for fulfilment.
- Number of certification aspects to address, such as compliance requirements and evidence artefacts to collect
- Overall duration and overall costs of the entire certification process (or phases of the entire certification process)

### 4.11 Randomness and security assessment process performance (VV-11)

Assessing the randomness and cryptographic strength (see Section 3.9) should be time- and effort-efficient as the cyber-physical systems to be validated and verified are complex systems and need to be restarted as soon as possible for their actual work. The V&V of cryptographic components is essential and should be tackled in the early phases of design, i.e., security-by-design. To improve the cyber-resilience of automated systems, the key generation/distribution mechanisms and cryptographic functions should be tested throughout the development process. Here, key generation schemes play a crucial role as the randomness, unpredictability, reproducibility, and robustness of true random number generators should be implemented. Automated or semi-automated methodologies are required to conduct the vulnerability analysis of security components, e.g. cryptographic hardware, mainly focusing on the crypto-key generation aligned with the architecture of common components of a typical cyber-physical system.

Moreover, the employment of less personnel effort is also crucial to improve labour efficiency as the proposed method will enable the verification of the crypto-key generation scheme by-design that will lead the overall design to a more resilient system. Thus, the randomness and security assessment tests are needed to be performed as fast and efficiently as possible, and the security assessments should be repeated even in actual running mode (e.g., monitor the generated bit strings and apply vulnerability analysis regularly on-the-fly).

VV-11 is followed in the scope of assessing the security of the quality inspection system used in automotive manufacturing processes. As improved according to its first version, the quality inspection system is designed to enable multistakeholder collaboration and access from online nodes. Thus, this upgrade brings additional security assessment that should be repeated regularly throughout the automotive body parts manufacturing life cycle. VV-11 is used to measure the total time needed to complete the regular security checks, especially the secret generation scheme used in IoT gateways (responsible for system data transfers to an online service), encryption of the critical data about the quality inspection system and the assessment of the person and node authentication. The cost estimation might be calculated as a function of time, the number of authorised personnel and their salaries, and the cost of maintenance and supply costs, e.g. consumables, devices, overheads, etc.

**Measured artefacts** Person-hours spent by the expert, and other costs related to the official certification of the secure components (e.g., common criteria evaluation, see Section 4.10).

In addition to the standards related to randomness, cryptographic strength, and authentication (e.g., NIST-800-22 ^50^, ISO/IEC 9798 ^41^, ISO/IEC 15408 ^42^, FIDO ^40^, etc.), the measuring the performance of the security assessment process is addressed in ISO/IEC 33073 ^88^. The proposed standard describes a Process Assessment Model (PAM) to perform a conformant assessment of the process capability in accordance with the security requirements in a typical cyber system.

### 4.12 Effort required by the user to prepare and run a V&V tool (VV-12)

This metric expresses the effort required by the user (on average) to set up and run a V&V tool to follow safety or cyber-security process defined in related standards such as ISO 25119 ^31^, or ISO 21434 ^32^. Each type of tool may require a different amount of effort from the user to allow the proper set-up and running of the application.

**Measured artefacts** Person-hours spent by the user for setting up and running the tool (it includes learning, configuration, customisation, preparation phases, and reuse).

### 4.13 Reliability measures of decisions (VV-13)

One of the main tasks of decision-making systems is to classify the situation, e.g., the actual status of the system or a system component, properly. Automatic V&V checkers are considered as such decision-making systems which classify the design, implementation, or behavioural status of a SUE if it fulfils the specified requirements. These checkers mostly operate not on real systems under test but on their models or virtual prototypes. If they decide that the analysed system contains a bug, such a decision can be wrong because the model of the SUE abstracts away some of the details of a real SUE behaviour. Even if the checker concludes that the SUE is correct, such a decision can also be wrong for the same reason. Thus, for every decision of automated checkers, there should be a proper confirmation. Unfortunately, the confirmation is mainly performed manually by an expert, which requires special effort and raises the development cost. That is why researchers focus not just on more precise models of SUE but also on more types of bugs to be revealed by their tools.

V&V checkers, so-called analysis tools or simply analysers, often conclude with the report of several issues they "think" need to be fixed in the developed SUE. The performance of such an analysis can be indicated by different statistical ratios calculating whether these decisions are correct or not. Similar to the evaluation criterion for AI/ML algorithms (see Section 3.4), decisions can be classified as true or false (if they are correct or not) as well as positives or negatives (if they indicate faults or correct behaviour). A true conclusion means that the decision is correct, a false conclusion means that an analyser is wrong. Positive (or sometimes called "alarm") means that the situation indicates a fault or a bug in the system; negative represents a correct behaviour:

- TP = true positive/alarm (analyser found a real bug in a system),
- FP = false positive/alarm (analyser reported a bug which is not a real issue in the system),
- TN = true negative (analyser did not find anything wrong on a correct artefact of SUT),
- FN = false negative (analyser did not find a bug in a faulty artefact of SUT).

Note that negatives in general are not reported at all. Analysers are mostly used for bug hunting and reporting possible bugs. Since analysers operate on source codes (e.g., static analysis of source code), automaton-based models, or executions of a system (runtime verification and dynamic analysis), it is hard to list all possible artefacts under study.

For instance, some static analysers search for possible data races in a program. Artefacts studied by the analyser are all memory accesses in a program, but some of them can be classified as data races, and some of them are regarded as correct ones. All data races that are recognised by an analyser are reported and can be further confirmed by a developer if they are valid or benign (i.e. if they are true or false positives). But no one can tell, how many other memory accesses are indeed correct or incorrect. That is why the number of false or true negatives is sometimes hard to say.

Even though there are standards which specify requirements for the assessment of tools and methods used in the development process, such as ISO 26262 ^13^ and RTCA/DO-330 ^89^, the standards do not specifically define metrics which are suitable for evaluation and/or selection of analysers. For that purpose, statistical ratios of true/false positives/negatives are commonly used for the evaluation of analysers. Such ratios include Accuracy, Precision, Recall, and False discovery rate:

- Accuracy (the higher the better): It indicates the overall conformance of the checker. All incorrect decisions (something good is reported as a bug or some real bug is not reported) are included. $Accuracy=\frac{\text{Number}\;\text{of}\;\text{correct}\;\text{decisions}}{\text{Total}\;\text{number}\;\text{of}\;\text{situations}}=\frac{TP+TN}{TP+TN+FP+FN}$ . Accuracy = 1.0 means the verification method is sound and complete.
- Precision (the higher the better): It indicates the proportion of correct positive identifications: $Precision=\frac{TP}{TP+FP}$ . Precision = 1.0 means the verification method is complete (all alarms are valid).
- Recall (the higher the better): It indicates the proportion of actual positives that are correctly identified: $Recall=\frac{TP}{TP+FN}$ . Recall = 1.0 means the verification method is sound (i.e., no potential bug is missed).
- False discovery rate (FDR, the lower the better): It specifies the possibility of alarms reported being incorrect. FDR is an inverse indicator of precision. $FDR=\frac{FP}{FP+TP}$ .

Static code analysers based on formal methods mostly overapproximate the behaviour of a program under test in order to be safe with the conclusion (i.e., no potential bug is silenced). Their Precision then equals to 1 but the alarms studied afterwards increase the time for debugging and their confirmation and if their FDR (False Discovery Rate) is high, developers tend not to use the analyser at all. Runtime monitors, on the other hand, when bugs are manifested during the execution of the SUT, report only true positives. The Recall of these tools equals 1. Unfortunately, no runtime analysis can ensure that no other bugs are present in the system (Precision is lower than 1).

**Measured artefacts** Number of all tests, number of true/false positives/negatives.

## 5 Experimental evaluation

**Figure 1.**
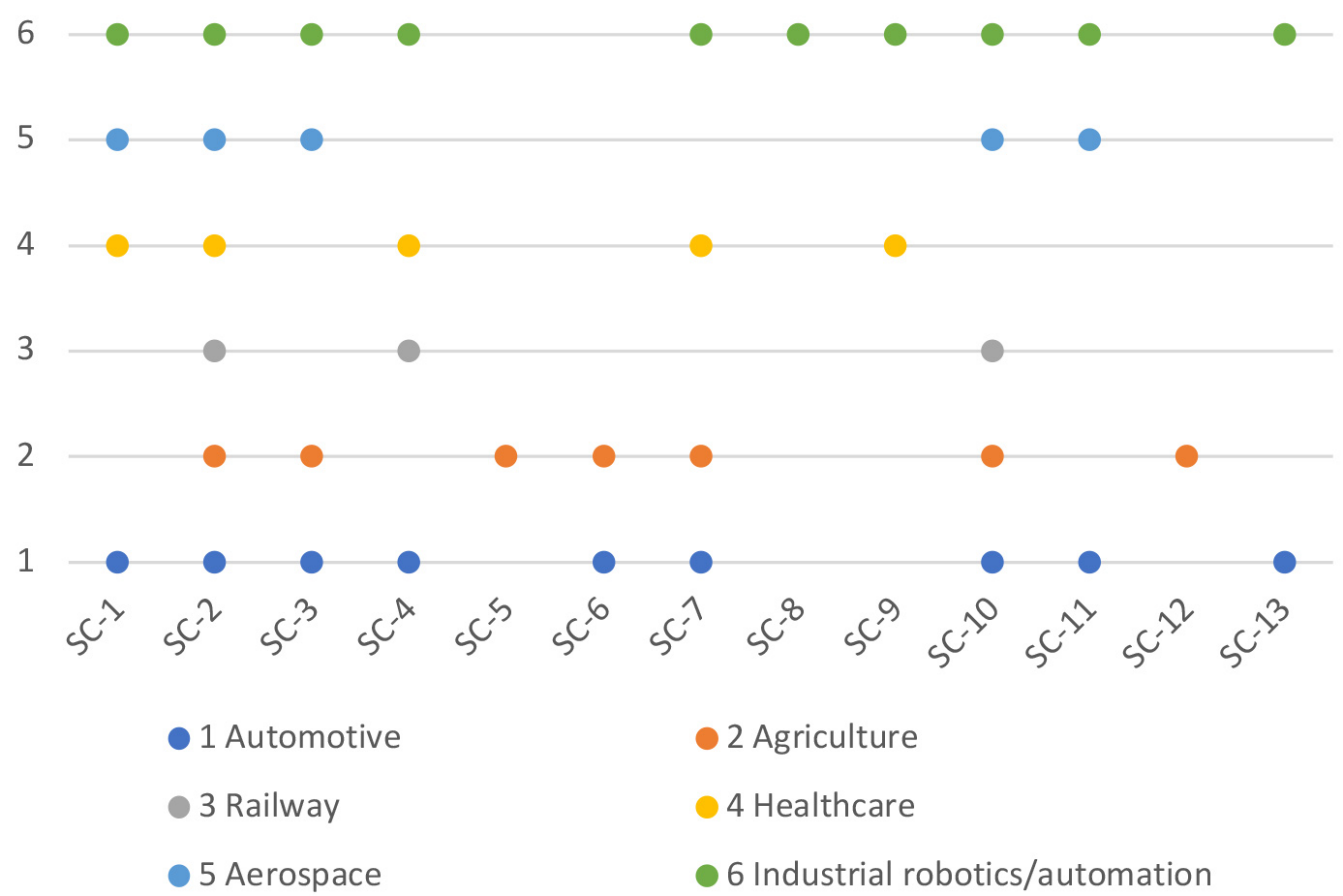
Mapping of SC evaluation criteria to the domains.

**Figure 2.**
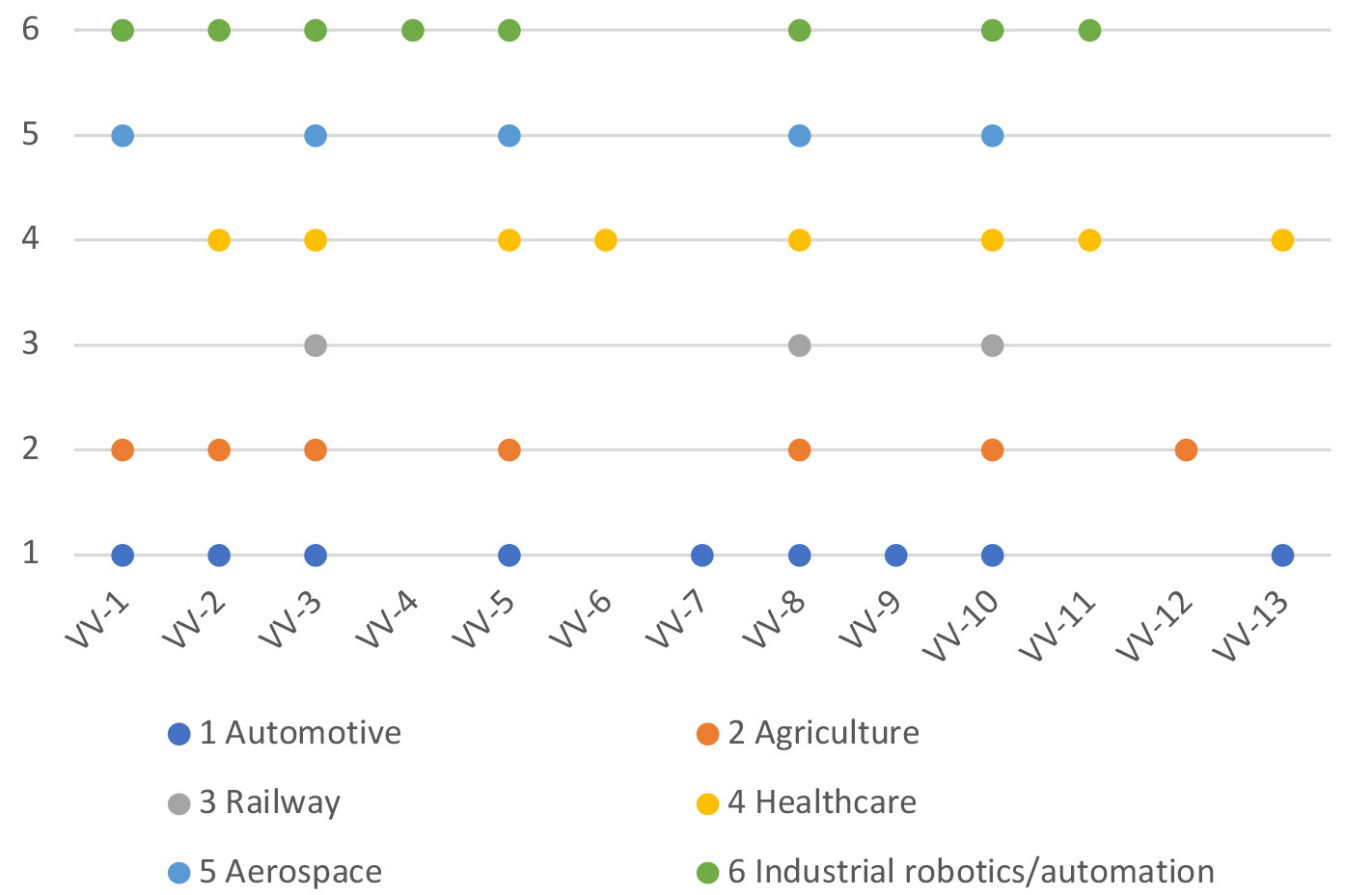
Mapping of V&V evaluation criteria to the domains.

The evaluation criteria presented in Section 3 and Section 4 are used to evaluate the 13 use cases presented in Table 1. To show the commonality in using the criteria across the domains for which the use cases are developed in, we mapped the evaluation criteria to 6 domains of interest. Figure 1 shows the mapping of the evaluation criteria for safety and cybersecurity to these domains, and Figure 2 presents the mapping of the V&V evaluation criteria to the domains. The criteria are identified with SC and VV following the number of the criterion. Note that the figures provide a summary of the usefulness of the criteria in specific domains, however, the criteria could be used by practitioners in any other use cases and domains. For instance, a remotely-controlled agriculture robot shares similar features with a teleoperated car, but they might deal with different cybersecurity and safety evaluation criteria due to different regulations and environmental conditions in which the systems will be operated.

To show the usefulness of the criteria proposed, and in the remainder of the paper, we present concrete quantitative results obtained from the usage of the criteria in several use cases. The results could be found in Table 2 and Table 3. Note that the results here are presented at a high level, which is in line with the evaluation goal of this paper. For additional details about the results obtained as well as the evaluation setup used, we encourage interested readers to take a look at the references to scientific papers published that are provided in these tables. Moreover, several other scientific articles are under preparation where additional details about the evaluation results are presented. Upon their publication, these scientific articles will also be uploaded to the VALU3S project website ^7^.

**Table 2.** Evaluation using the criteria for safety and cybersecurity.

Criteria	Value	Description
SC-1	24% error coverage	This criterion has been used to measure the impact of InvertMessage attacks on a Simulink model of a break-by-wire system. After conducting 691 experiments using this attack model, the error coverage with respect to the safety requirement defined was measured to be about 24%. Note that, the reason for such low error coverage is that the system under test was not equipped with any safety mechanisms. More information about the experimental setup as well as the results obtained could be found in ^65^.
SC-2	0 violations but the number of requirements increased from 14 to 42 during formalisation	The aerospace use case, which is focused on an aircraft engine software controller, has not yet detected specific requirement violations (although work is ongoing). However, much time was spent in accurately eliciting and formalising the original set of natural language requirements. There were 14 requirements originally, and, after a thorough elicitation process, this number increased to 42 requirements ^30^. This demonstrates that significant ambiguities were present in the natural-language requirements that could be identified and captured by formalising the requirements.
SC-3	*DR*_*attack*_ target > 95%	For instance, in the development of a remotely-controlled agricultural robot, this criterion can be used to quantify the number of malicious attacks performed as penetration tests among the ones carried out that have been successfully detected and blocked so as to not cause any malfunction to the attacked robot (including taking physical control of the robot).
SC-4	99.7% Accuracy	A vision-based vehicle identification system, which uses low-quality images captured by a monocular video camera mounted at the front of the car, is based on different AI systems including convolutional neural networks (CNN), algorithms based on the histogram of oriented gradient (HOG), or a technique using a support vector machine (SVM). The accuracy of detection using CNN is up to 99.7% while other techniques of the same conditions have the accuracy 94.88% (CR-HOG) or 91.2% (HOG+SVM) ^90^.
SC-5	System robust against attacks from Level 2 to Level 5	For instance, in the development of a remotely-controlled cyber-physical system, this criterion can be used to evaluate the potential impact of the implemented attacks on the normal operations of the attacked system.
SC-6	40% increase	When analysing threats and assessing security risks, it has been shown that visual methods can be more effective in threat identification than textual ones ^91^, leading to an increase of identified threats
SC-7	55% and 45% prevented accidents	This criterion has been used to measure the impact of five Jamming attacks in a platoon of four vehicles ^21^. The jamming attacks investigated are modelled using ComFASE (a Communication Fault and Attack Simulation Engine) ^20^ and represent three real-world attacks, namely, *destructive interference*, *barrage jamming*, and *deceptive jamming*. The attacks are injected in the physical layer of the IEEE 802.11p communication protocol simulated in Veins (a vehicular network simulator) ^92^. To understand the number of accidents that could have been prevented, here we present some additional details about the destructive interference experiments conducted on the 2nd vehicle in the platoon. Out of the 33,750 experiments conducted, 8,827 resulted in accidents. In this study, the authors have not investigated the extent to which safety mechanisms could have prevented these accidents from happening. However, if the vehicles were equipped with special sensors, 1,325 frontal and 1,108 rear-end accidents could have been prevented. More information about the experimental setup as well as the results obtained could be found in ^21^.
SC-8	Person Authentication: Accuracy 99.56%, Average duration: 1.23 seconds; Node authentication: Accuracy 99.99%, Average duration: 125 milliseconds.	This criterion has been used to evaluate the authentication of authorised users to access the automotive quality inspection system in a factory setting. 25 subjects are asked to try the authentication token and the FIDO-compliant Authentication-as-a-Service. Node authentication is applied to the secure IoT gateway, considering this device as a node, which is connected to the main control unit of the robotic system (that is composed of cameras, robotic arms on which cameras are mounted, control units and the data management system) used in the VALU3S use cases.
SC-9	Fulfil 100% of the NIST-800-22 True randomness test criteria; AES Speed: (128 bit) 1 Gibt/sec with 250 MHz frequency and 32 clock cycle; 3DES Speed: 900 Mbit/sec with 250 MHz frequency and 17 clock cycles; Encryption duration:10 milliseconds for 1 KB images.	This criterion has been used in the automotive quality inspection system in VALU3S to enable the encryption and secure transmission of the images captured by the robotic system used in automotive parts quality control. The captured images are transmitted to third-party software, namely the Camera Fault Injection and Anomaly Detection tool, over a secureWeb channel. The cryptographic backend is managed by PRIGM (trademark by ERARGE), a hardware security module, at the server side which employs true random number generators. A secure gateway is mounted at the edge side (on the robotic quality inspection system at the factory side) enabling end-to-end security.
SC-10	FTR = 13.7%	In a railway interlocking system composed of several sub-systems distributed geographically, a specification of a motor controller identifies 146 behavioural faults. Improper manual coding covers 20 faults, which would lead to 13.7% fault tolerance and robustness.
SC-11	SystemRobustness = 91.98%	The industrial robotics system’s robustness was evaluated using a specific criterion. Out of 3381 tests carried out, the system successfully identified faults in 2839 instances. However, the system did not perform accurately in 271 tests on 22 distinct files within the system. When considering the 2839 successful test outcomes, the SystemRobustness is computed to be 91.98.%.
SC-12	100% of the hazards of ^57^	This criterion is used to define the percentage of hazardous event typologies examined by a risk assessment tool developed in the VALU3S project
SC-13	7 functionalities of a simulator including a real-time rendering (28.5 million polygons, 68 fps) compared to original 2 test conditions	Simulator environment of 3D scenes used for generating test inputs for intelligent surveillance system implements 6 different weather conditions. Moreover, a significant increase in the number of polygons in a simulated scene while keeping rendering performance above 60 fps allows real-time testing of the intelligent surveillance system. The testing of the surveillance system used a limited number of static images capturing real situations of 2 different weather conditions. Unreal Engine substantially enhanced the quality of 3D scenes enabling real-time testing of a large number of different situations.

**Table 3.** Evaluation using the criteria for for measuring the efficiency of V&V processes.

Criteria	Value	Description
VV-1	From 60 to 1 minute (98% reduction)	CardioWheel is an Advanced Driver Assistance System that acquires electrocardiogram from a driver’s hands to continuously detect drowsiness, cardiac health problems, and perform biometric identity recognition. Hardware, firmware, and signals need to be tested for each new implementation of the system. After automating test execution, the time required for system testing has decreased from up to 60 minutes to around 1 minute.
VV-2	From 56% to 76% statement coverage	Measuring software artefacts covered by tests is crucial feedback on how well the system has been tested. The module which manages a safe and secure link between the vehicle and the remote station is one of the critical parts of the system for a remotely operated car. Six automated tests of simulated driving verify behaviour in different situations and cover up to 56% of source-code statements. Incorporating fault injection of a network link during testing increases the statement coverage up to 76%.
VV-3	30% test space reduction	This criterion has been used to measure the amount of reduction in the size of the test space when incorporating a test space pruning technique called *error space pruning of signals* ^65^. This technique works by considering attacks on input signals to be equivalent to those on an output signal if only one propagation path exists between the input and output signal. The technique has been Incorporated into MODIFI ^93^, a fault and attack injection engine suitable for evaluating MATLAB Simulink models. After applying the technique to a Simulink model of a break-by-wire system, we reduced the size of the test space by 30%. More information about the experimental setup as well as the results obtained could be found in ^65^.
VV-4	From 20 minutes to 7 minutes per test case in average (65% reduction)	Designing a simple test case for a manufacturing execution system (MES), which tests a manufacturing technology and communication between MES and a machine, takes approximately 10–30 minutes. The test design is manual and feature-driven. The total time spent depends on the complexity of the test. Automated test case generation exploiting model-based techniques takes 20–60 minutes. This includes manual creation of the test case in the same way as before, plus formalising the test case but generating up to 6 test cases. The average time before: 20 minutes per test case; time after: 40 minutes for 6 test cases, about 7 minutes per test case.
VV-5	251,250 test cases	This criterion has been used to measure the impact of five Jamming attacks in a platoon of four vehicles (see SCP-7 in Table 2). The total number of experiments (test cases) executed is 251,250. All these test cases are created to analyze the implication of cybersecurity attacks on system safety. In fact, 92,036 of these test cases resulted in collision incidents which directly translated into a violation of system safety. More information about the experimental setup as well as the results obtained could be found in ^21^.
VV-6	From 9 hours to 3 hours	When testing a system for remote control of a vehicle, the estimated time for manual preparation of 6 simple tests is 6 hours. Manual test execution time takes 10 minutes for each test. Finding and fixing a bug takes an average of 2 hours in such scenarios where a simulation model replaces a real vehicle. The cost of a bug found using manual testing is 9 hours. Automation reduces the time for test preparation (10 minutes per test) and test execution (1 minute per test). Moreover, it can uncover more bugs in the system’s future development. The cost of a bug is approximately 3 hours.
VV-7	99.7% test execution coverage in a week	Manufacturing execution system (MES) consists of a large number of specific-purpose software components developed separately. Regular regression tests for every updated component and code reviews are required to monitor the quality of the MES development. For example, a weekly run pipeline for building the whole MES using the Jenkins automation tool reported 371 passed test suites and 2 not executed regression test suites.
VV-8	From 26 minutes to 19 minutes (27% effort saving)	This criterion has been used to measure the efforts the industrial robotics system saved in performing quality control inspections for body-in-white. Initially, it took the robots 26 minutes to complete the operations. By using V&V operations in accordance with ISO 15288 standards and the fault injection method, the time was reduced to 19 minutes, resulting in a 27% effort saving.
VV-9	40% action reduction	It has been reported that systematic engineering practices, including V&V ones, for embedded software can reduce maintenance costs by more than 40% ^94^, thus the effort.
VV-10	54% effort reduction and 27% cost reduction	When reusing a safety-critical product, and by following a systematic approach to assess V&V needs and reuse consequences, it has been estimated that (re-)certification effort and cost can be reduced thanks to the reuse of certification information related to the criteria to comply with and the evidence artefacts to provide ^95^.
VV-11	Average expert effort: 3 hours	This criterion has been used to evaluate the potential vulnerabilities of a random number generator that can be used in the robotic automotive quality inspection system or other cyber-physical systems addressed in other VALU3S use cases. Random number generators are one of the most critical components of a cryptosystem as they are used to generate cryptographic keys, secrets and one-time-passwords (used for node or person authentication). The true randomness criteria are critical but not sufficient as there is a strong need for an expert opinion to verify if the developed hardware-based TRNG generates unpredictable and irreproducible bit streams that rely on robust hardware design. VV-10 shortens this process to less than 3 hours according to our technical discussions with 5 experts, where we ask them to verify the random number sequences generated by a typical Arduino Uno Board and ERARGE’s ring-oscillator-based and chaotic TRNGs.
VV-12	50% effort reduction	This criterion is used to evaluate the effort reduction to perform risk assessment using a risk assessment tool such as CHESS-FLA compared to risk assessment done using a spreadsheet. A risk assessment tool requires an effort spent on designing a system functional model and failure behaviour but can invoke a fully automated failure logical analysis. For a system with a complexity similar to a robotic lawn mower, the effort reduction is about 50%.
VV-13	Accuracy improvement from 65% to 77%	Data-race detectors in dynamic analysers of software are based on different detection algorithms which may produce false alarms or miss the error. The FastTrack algorithm, which motivates the implementation of a well-known Valgrind/Helgrind tool, performs quite well (Precision 0.67, Recall 0.95), yet the Accuracy is quite low: 0.65. The method enhanced with noise injection increased Accuracy to 0.77 ^96^.

## 6 Conclusion

Verification and validation (V&V) of a cyber-physical system (CPS) are key processes needed to build safe and secure systems with high levels of criticality. V&V methods used on CPSs must handle both computation and physical processes and their mutual influence. Practitioners of CPS have plenty of V&V methods, tools, and toolchains to choose from. Their selection depends on the technology features, how the technology helps with high levels of assurance, and the impact of the technology on the product life-cycle processes.

To address these needs, we have presented a set of thirteen criteria for the measurement of safety and cybersecurity of the CPS as well as thirteen criteria aiming at the measurement of V&V processes. The criteria have been collected by 60 practitioners, researchers and technology providers jointly working on 13 real-world use cases from different domains. The extensive set of criteria targets use cases focusing on the development of products with various technology readiness levels, various levels of criticality, and various V&V processes within organisations.

We concluded that the most common criterion is SC-2 measuring how many requirements have been violated. The criterion follows the current practice in almost any CPS development as one of the earliest stages of development includes requirements analysis. Another commonly used criterion is SC-7 which is suitable for measuring the improvement of the safety of CPS. If an autonomous CPS include artificial intelligence or machine learning, the used algorithms are often evaluated using the metrics selected from criterion SC-4. Considering the spent time and cost on V&V, the most common criteria are VV-3, VV-8, and VV-10. These criteria focus on testing and certification, which are integral parts of the process of CPS development.

In further studies, qualitative criteria are planned to be added to the quantitative metrics listed in this paper. These metrics will be assessed and reported with real-life use cases and field demonstrators for industry-driven applications and future research activities.

## Data Availability

All data underlying the results are available as part of the article and no additional source data are required.
