# Supplementary material for: Towards an extensive set of criteria for safety and cyber-security evaluation of cyber-physical systems
Source: Open Res Eur. 2023 Dec 28;3:219. [Version 1] doi: 10.12688/openreseurope.16234.1 (PMC11026953; doi:10.12688/openreseurope.16234.1)
Supplement: Peer review history

# Reviewer report: Reviewer response for version 1

**DOI:** 10.21956/openreseurope.17525.r37590

**Published:** 2024-04-18

**Authors:** Simona Bernardi

**Affiliations:** 1 University of Zaragoza, Zaragoza, Spain

The paper presents a set of criteria for the evaluation of the verification and validation (V&V) activities aimed at the safety and cybersecurity assessment in cyber-physical (CPS) system domain. The criteria are organized in two categories: 1) metrics for the evaluation of safety and cybersecurity of CPS; and 2) criteria for the evaluation of the V&V activities themselves.

 The criteria have been defined within a large-scale European project, considering a notable number of case studies (13 case studies) from different CPS domains.

 The paper provides a valuable contribution toward the setting of a common baseline for the assessment of V&V safety and cybersecurity activities. Most of the criteria are common to different case studies, which is a good indicator for generalization purpose.

 The paper is easy to read, however the organization of the material could be improved.

 In particular, I missed the rationale in the presentation of the different criteria, that is the approach followed to come to the set of criteria and the order they are presented.

 Concerning the criteria in the first set, I suggest to use the same structure for the presentation

 of each one (possible a table that summarizes them could be added): indeed, for all the criteria

 metrics are presented but just in few cases also "acceptable criteria" are added (without referencing the concrete cases where they are considered acceptable, e.g., SC-8.SC-9).

 I also suggest to revise the presentation of the following criteria: SC-4, SC-8, SC-9 (detailed comment below).

*Detailed comments:*

 - Metrics to evaluate artificial intelligence/machine learning algorithms (SC-4)

 1) The TP/TN/FP/FN metrics (confusion matrix) are the basic metrics that usually are not used directly to assess a ML techniques but are the ones the usual "performance" metrics rely on (accuracy, precision, recall etc.).

 Thus, possibly they could be presented first and separately from the derived ones.

 2) The following sentence:

 "Furthermore, specific domains may impose specific preferences, for example, measuring accuracy may suffice for many automotive applications, while healthcare scenarios most often require measuring sensitivity and specificity."

 maybe misleading and could better stated, since the choice of a metric should also depend on the  data and the how the testing experiments are carried out (e.g., if an experiment to test the performance of a ML technique is based on unbalanced dataset, the accuracy is not an adequate metric, i.e., provide unfair performance results).

 3) It is not very clear why the classification threshold is considered a metric, rather than a parameter of the classifier.

 - Authentication accuracy and time applied to human users and components (SC-8)

 The acceptance criteria are not clearly defined.

 In particular:

 1) What is the meaning of "authentication accuracy rate"?

 2) How the "authentication duration in seconds" could be interpreted (and therefore used for the evaluation):  is it a real-time requirement or a soft real-time requirements?

 - Randomness and cryptographic algorithm strength (SC-9)

 The following sentence:

 "According to Kerckhoffs assumption although cryptographic algorithms are crucial and must be kept secret, the strength of a cryptosystem is highly dependent on the cryptographic key

 generation mechanism."

 could be revised since the Kerckhoffs' principle assumes the publicly availability of crypto-algorithms.

 In particular, it states that: "the security of a cryptosystem must lie in the choice of its keys only; everything else (including the algorithm itself) should be considered public knowledge."

Is the study design appropriate and does the work have academic merit?

Yes

Is the work clearly and accurately presented and does it cite the current literature?

Partly

If applicable, is the statistical analysis and its interpretation appropriate?

Not applicable

Are all the source data underlying the results available to ensure full reproducibility?

Yes

Are the conclusions drawn adequately supported by the results?

Yes

Are sufficient details of methods and analysis provided to allow replication by others?

Yes

Reviewer Expertise:

Model-driven engineering, V&V of non functional properties (performance, dependability, resilience)

I confirm that I have read this submission and believe that I have an appropriate level of expertise to confirm that it is of an acceptable scientific standard.

## References

[1] : Age Differences in Physiological Reactivity to Daily Emotional Experiences. *Affect Sci* .2023;4(3) : 10.1007/s42761-023-00207-z 487-499 10.1007/s42761-023-00207-z 37744978 PMC10514012

# Reviewer report: Reviewer response for version 1

**DOI:** 10.21956/openreseurope.17525.r37592

**Published:** 2024-02-09

**Authors:** António Casimiro

**Affiliations:** 1 University of Lisbon, Lisbon, Portugal

This paper provides a collection of metrics that may be used to assess safety and security aspects of cyber-physical systems. The collection is quite broad and the metrics are described in detail. Furthermore, the paper splits the metrics in two groups: one that focuses specifically on system-related metrics (i.e., metrics to measure system security and safety attributes), and another that focuses on how to measure the verification and validation process itself. Finally, the paper briefly addresses the application of the described metrics to a set of domains.

 Overall, the contribution of the paper is valuable, and will likely be relevant to many researchers and practitioners. The proposed systematization, which serves to organize the many metrics, makes sense and is sound, even if a different organization could be considered.

 What I felt was missing was the following.

 First, the set of metrics, which are organized as a set of criteria, is introduced too briefly, just referring to list of parameters/aspects that were considered. This should be improved to provide a rationale for how the sets were defined, that is, why they considered some requirements (e.g., requirements on ML performance) and not others, and to which extent the considered set is complete or is missing other requirements. 

 Second, it is very surprising that real-time requirements were completely ignored, especially because the paper is targeting cyber-physical systems, which almost always have real-time requirements (in many cases hard, safety-critical requirements). Therefore, I recommend that the paper is improved by adding criteria for the evaluation of time-related aspects, like to measure deadline misses, response time, etc.

Is the study design appropriate and does the work have academic merit?

Yes

Is the work clearly and accurately presented and does it cite the current literature?

Yes

If applicable, is the statistical analysis and its interpretation appropriate?

Not applicable

Are all the source data underlying the results available to ensure full reproducibility?

Yes

Are the conclusions drawn adequately supported by the results?

Yes

Are sufficient details of methods and analysis provided to allow replication by others?

Yes

Reviewer Expertise:

Distributed Systems and Cyber-Physical Systems Architectures, Dependability, Security, Real-Time

I confirm that I have read this submission and believe that I have an appropriate level of expertise to confirm that it is of an acceptable scientific standard.
